# Size-Controlled Silver Nanoparticles Supported by Pyrolytic Carbon from Microcrystalline Cellulose

**DOI:** 10.3390/ijms241914431

**Published:** 2023-09-22

**Authors:** Dayong Huang, Min Wu, Shigenori Kuga, Yong Huang

**Affiliations:** 1 National Engineering Research Center of Engineering Plastics, Technical Institute of Physics and Chemistry, Chinese Academy of Sciences, Beijing 100190, China; huangdayong14@mails.ucas.ac.cn (D.H.); skaggs@kfz.biglobe.ne.jp (S.K.); 2 Xiong’an Institute of Innovation, Xiong’an 071899, China; 3 Center of Materials Science and Opto-Electronic Technology, University of Chinese Academy of Sciences, Beijing 100049, China

**Keywords:** silver nanospheres, nanocomposite, cellulose, pyrolysis, antimicrobial

## Abstract

A facile method was developed for preparing size-controlled silver nanoparticles supported by pyrolytic carbon from microcrystalline cellulose (MCC). The pyrolysis of cellulose-AgNO_3_ mixture caused the oxidation of cellulose, resulting in carboxyl groups to which silver ions can bind firmly and act as nuclei for the deposition of silver nanoparticles. The structure and properties of the obtained nanocomposite were characterized by using a scanning electron microscope (SEM), transmission electron microscopy (TEM), thermogravimetric analysis (TGA), Fourier transform infrared (FT-IR) and X-ray diffraction (XRD). The results suggest that silver nanoparticles were integrated successfully and dispersed uniformly in the pyrolytic carbon matrix. The average particle size varied between 20 nm and 100 nm in correlation to the dose of silver nitrate and temperature of pyrolysis. The products showed high electric conductivity and strong antimicrobial activity against *Escherichia coli* (*E. coli*).

## 1. Introduction

Recently, there have been advances in nanomaterials, such as the development of metal nanoparticle-based materials for biomedical imaging and cancer therapy [1,2]. Silver nanomaterials such as spheres, rods and sheets attract considerable attention due to their potential as catalytic, electronic, optical and thermal materials [3,4,5,6,7,8,9,10,11]. Additionally, they work as antibacterial agents against numerous pathogens [12,13,14,15,16,17]. Various methods have been developed for the preparation of silver nanoparticles [18,19,20,21], mainly the chemical reduction of silver cation. The typical method is the use of silver nitrate and a reductant such as NaBH_4_, aldehydes, or polysaccharides [22,23,24]. While these methods are known to give various products in different sizes and shapes, they require rather accurate control of the reaction conditions and fine chemical reagents [25]. Furthermore, because of issues with aggregation and coagulation, it appears hard to directly use colloidal silver nanoparticles as a catalyst or as an anti-microbial agent.

The use of template material as a reaction medium is one method of tackling issues [26,27,28,29] with metal organic frameworks (MOFs), mesoporous silica, graphene, active carbon, polymers and ceramics. For example, Ag/Au nanoparticles encapsulated into MOFs can serve as active layers for electrical bistability devices [30]; mesoporous silica-coated Au-core Pt-shell as a nanoprobe for highly efficient virus diagnosis [31]; graphene oxide enwrapped Ag/AgX (X = Br, Cl) nanocomposite as a highly efficient visible-light plasmonic photocatalyst [32]; and turning fulvic acid into silver-loaded carbon nanosheet can serve as a regenerable sorbent for mercury removal [33]. The high cost of the aforementioned template materials makes large-scale applications challenging to implement. Consequently, it is crucial and still a big task to create high-performance silver nanocomposite at a reasonable cost using sustainable ingredients.

A promising substance, cellulose is nontoxic, biodegradable, renewable, and environmentally friendly [34,35,36]. There have been some reports regarding the application of cellulose as a template, including nanocellulose [37], bacterial cellulose [38] and cellulose derivatives [39] for metallic nanoparticles. Microcrystalline cellulose (MCC) is a purified, partially depolymerized cellulose consisting of porous particles, which are colorless, tasteless, low-cost and non-toxic. Therefore, MCC is widely used as medical excipient, food and cosmetics [40]. However, to the best of knowledge, using MCC as a template for silver nanoparticle growth has not been reported.

Immobilized metallic nanoparticles attached firmly on the surface of cellulose is crucial to fully taking advantage of the quantum size effects of metal nanoparticles. However, the interaction is too weak to anchor metallic ions on cellulose. Numerous studies have therefore focused on chemically altering cellulose to increase the number of interactions between metal ions and cellulose [41]. For instance, TEMPO-mediated oxidized bacterial cellulose nanofibers can be used as efficient templates to support metal nanoparticles because of the strong interactions between metal ions and the carboxylate group [41]. According to our previous research [42], dialdehyde cellulose microfibrils have previously been shown to be effective growth templates for silver nanoparticles. However, the employment of hazardous reagents in the chemical modifying process inevitably causes a negative effect on the environment. Therefore, to promote the large-scale preparation of silver nanocomposite and alleviate the pollution of the environment, it is desirable to reduce silver ions in situ on the surface of templates by a facile method without using hazardous reagent [43,44,45].

In this study, we developed the use of MCC as a template-cum-carbon precursor to facilitate the manufacturing of silver nanoparticles, supported by an active substrate for versatility. MCC is a long-chain linear polysaccharide polymer of glucose monomers that are joined by β-1,4-glycosidic bonds, and its surface contains a large number of hydroxyl groups. Those -OH groups have a strong interaction with Ag^+^ by electrostatic force and could be oxidized to carboxyl groups in the process of pyrolysis. More significantly, because the carboxyl groups are more electronegative than hydroxyl ones, the oxidized cellulose has a higher adsorption impact on silver ions. Based on their chemical nature, both cellulose and pyrolytic carbon can act as reductant for silver cation. The pyrolysis of cellulose with impregnated silver nitrate can cause oxidization and carbonization of cellulose and reduction of silver in one step, providing a facile process to produce novel silver nanomaterials.

## 2. Results and Discussion

### 2.1. Silver Nanosphere Formation by Pyrolysis

**Figure 1:**
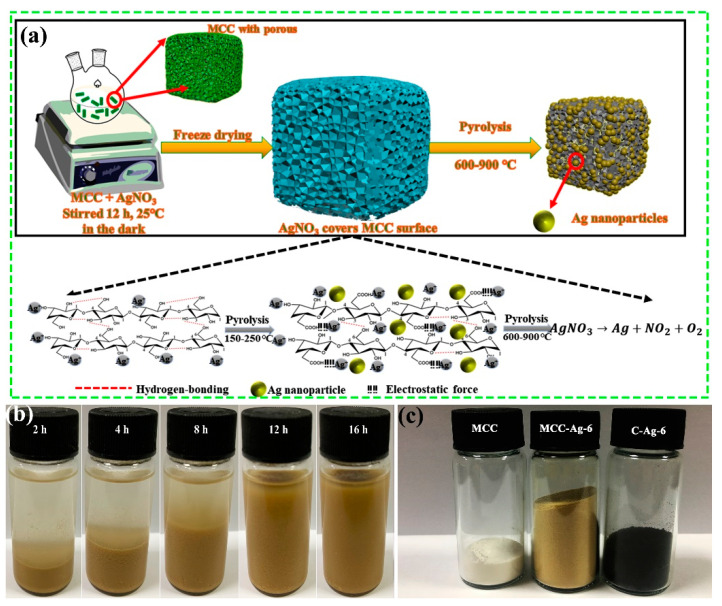
(**a**) Schematic program of preparation process of silver/carbon nanocomposite; (**b**) picture of MCC-Ag-6 with different swelling time; (**c**) picture of the samples of MCC, MCC-Ag-6 and C-Ag-6, the mass of all samples is 1.0 g, and the volume of the bottle is 10 mL.

Figure 1a depicts the steps involved in creating silver nanoparticles using MCC as a template. The 5 g of microcrystalline cellulose dispersed in 100 mL AgNO_3_ solution at first formed a sediment layer of about 1/5 of its height. Then, the layer gradually absorbed the solution by swelling and finally occupied the entire volume after 12 h standing at room temperature (Figure 1b). It is worth noting that the sample colour will be changed from white to yellow–brown as reaction time increases. This behaviour suggested that MCC may be oxidised by silver ions following a prolonged reaction (the details of which are included in the subsequent FT-IR study results). The freezing of MCC-AgNO_3_ dispersions quickly with liquid nitrogen can immobilize the silver nitrate in the pores of the MCC uniformly. Freeze drying this wet mass produced a yellow–brownish fluffy powder (MCC-Ag-6), which turned into a black mass (Figure 1c) after pyrolysis (C-Ag-6).

**Figure 2:**
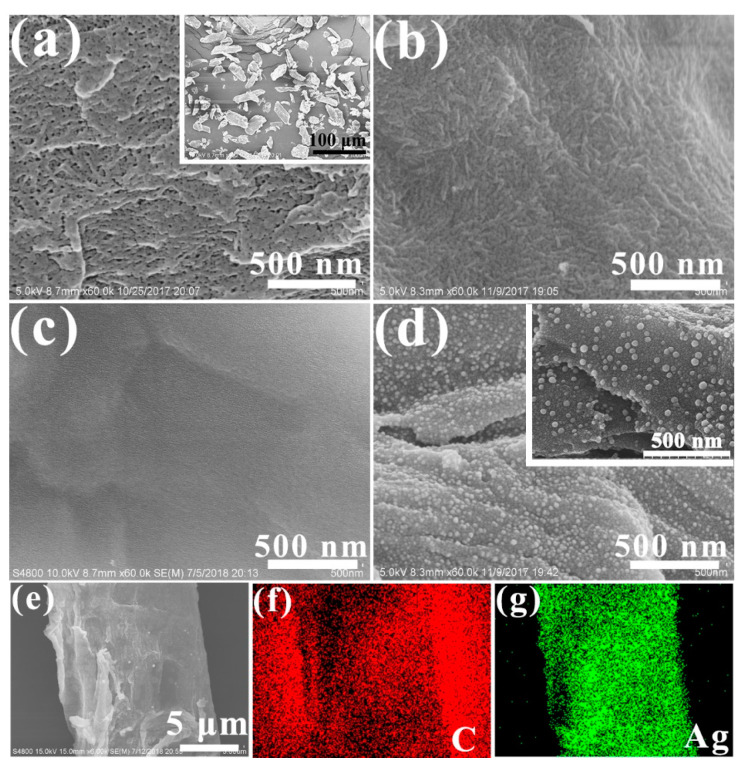
SEM images of (**a**) MCC, inset is low-magnification image; (**b**) MCC-Ag-6, (**c**) Pyrolytic carbon from pure cellulose; (**d**) C-Ag-6-600, inset is cracked face; (**e**) SEM image of C-Ag-6 and corresponding elemental mapping of (**f**) C and (**g**) Ag.

The surface morphology of the MCC and the size of the silver nanoparticles inside carbon matrix were observed by the SEM and TEM. Figure 2 shows the SEM images of the raw material and the pyrolysis product. The MCC consists of irregularly shaped cotton fibre fragments with sizes ranging from 10 um–50 um (Figure 2a inset). Its surface has many nanopores formed by the stacking of elementary fibrils (Figure 2a). The smooth surface of the AgNO_3_-infused freeze-dried cellulose (MCC-Ag-6) was devoid of any particles or pores, showing that AgNO_3_ had been uniformly impregnated into the swelling cellulose particles (Figure 2b).

The pyrolytic carbon from pure cellulose (Figure 2c) had a smooth surface as a result of pyrolytic carbonization of cellulose. In contrast, the carbon from MCC-Ag-6 was covered all over by 10–40 nm wide particles, apparently those of metallic silver formed by the reduction of silver (Figure 2d), as shown by the EDS element mapping (Figure 2g). These results further prove the following points: (i) freeze drying is an effective approach for impregnating AgNO_3_ on to the pores of MCC uniformly; (ii) the pyrolysis of cellulose-AgNO_3_ mixture will introduce a strong interaction with silver ions, so the silver nanoparticles can be firmly deposited into the pores of the MCC.

**Figure 3:**
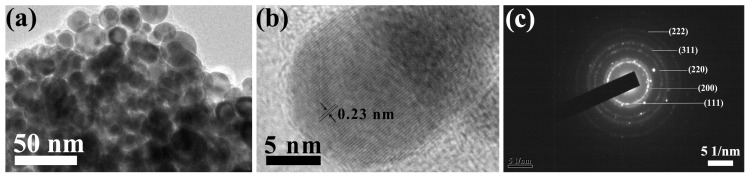
TEM images of silver particles in C-Ag-6 (**a**). Lattice image (**b**) and diffraction are those of cubic silver crystal (**c**).

Figure 3 shows TEM images of the silver particles in C-Ag-6. The particle shapes and sizes are identical to those in the SEM. TEM also produced lattice images and electron diffraction patterns of silver crystallites (Figure 3b,c). Electron diffraction (Figure 3c) shows a clear pattern of five rings of the cubic crystal of silver [46,47]. There is no doubt that the silver nanoparticles were well crystallized.

**Figure 4:**
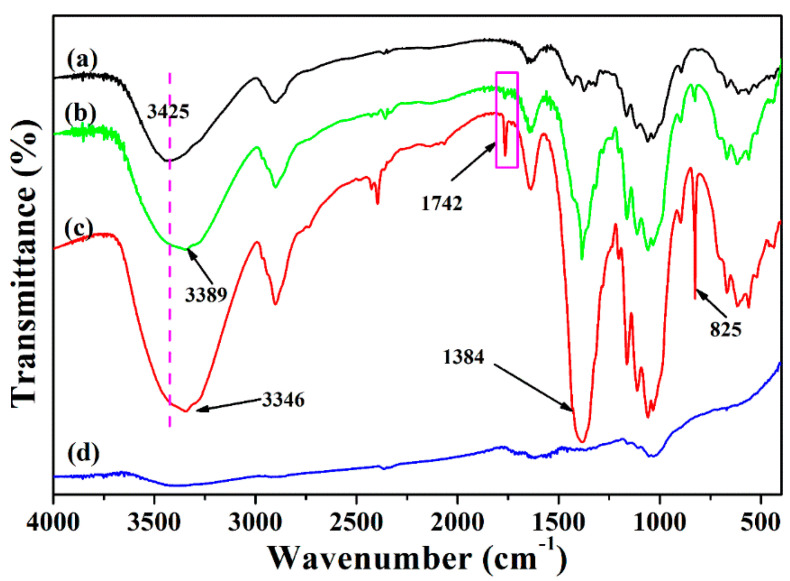
FT-IR spectra of (a) MCC; (b) MCC-Ag-6; (c) MCC-Ag-6 treated at 150 °C for 10 min under nitrogen; and (d) C-Ag-6.

The FT-IR was used to further confirm the reaction taking place throughout the pyrolysis process. Figure 4 shows FT-IR spectra of MCC, MCC-Ag-6 and MCC-Ag-6 treated at 150 °C and C-Ag-6. Original MCC has no adsorption peak band from 1700 to 1800 cm^−1^ (Figure 4a). In Figure 4b,c, the new peaks at 825, 1384 and 1724 cm^−1^ were introduced by the AgNO_3_. The adsorption bands at 825 and 1384 cm^−1^ can be assigned to the Ag-O and N-O group, respectively. The band at 1724 cm^−1^, which is likely due to the C=O stretching vibration of the carboxylic acid group, indicated that the hydroxyl group of cellulose was oxidized to the carboxyl group [48]. The peak intensity of MCC-Ag-6 at 1724 cm^−1^ is very weak, which means that silver nitrate can only partially oxidize cellulose at room temperature. However, after the thermal treatment of MCC-Ag-6 at 150 °C for 10 min, the peak intensity at 1724 cm^−1^ became stronger, suggesting that increasing the temperature is beneficial to the oxidation of cellulose by silver nitrate. A greater interaction between MCC and silver ions will occur as the temperature of the cellulose during the pyrolysis process rises, allowing the silver nanoparticles to eventually be securely impregnated on the carbon matrix. Comparing with MCC-Ag-6, the bands of C-Ag-6 at 825, 1384 and 3346 cm^−1^ disappeared, implying that the silver nitrate was converted to silver and the MCC was carbonized into carbon at 600 °C.

**Figure 5:**
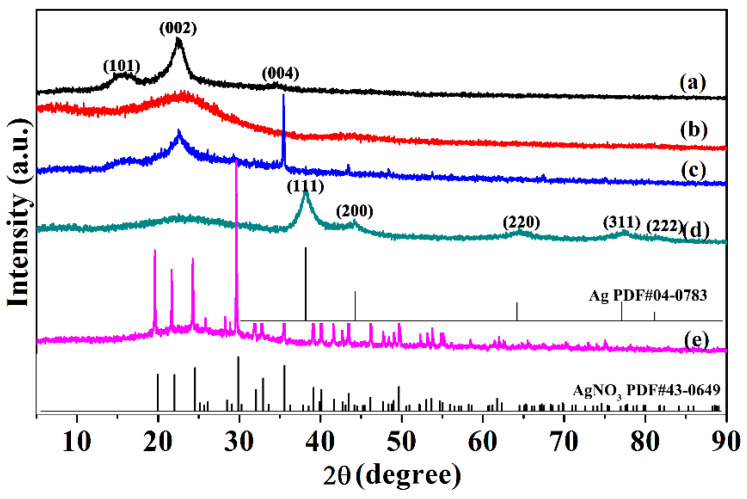
XRD patterns of (a) MCC; (b) pyrolytic carbon from MCC without adding AgNO_3_; (c) MCC/AgNO_3_ mixture; (d) C-Ag-6; and (e) AgNO_3_ powder.

Figure 5 shows the X-ray diffraction patterns of the products. The pattern of the MCC (Figure 5a) was that of cellulose Ⅰ. The pyrolysis of MCC at 600 °C converted the MCC into amorphous carbon (Figure 5b). Cellulose impregnated with AgNO_3_ (Figure 5c) produced many diffraction lines from the latter, and the cellulose diffraction lines were diminished due to oxidation by AgNO_3_. However, the diffraction line of the silver from the pattern of MCC-Ag-6 was not observed, which may be due to the limited ability of cellulose to reduce silver ions, so only a small portion of the silver ions were reduced. Therefore, it is necessary to pyrolyze MCC-Ag-x for the purpose of improving the yield of silver nanoparticles. The pyrolytic carbon from C-Ag-6 produced diffraction lines from graphite plus those of metallic silver [46,47]. (Figure 5d). It is indicated that the pyrolysis of cellulose with impregnated silver nitrate can cause the carbonization of cellulose and reduction of silver in one step with high efficiency.

**Figure 6:**
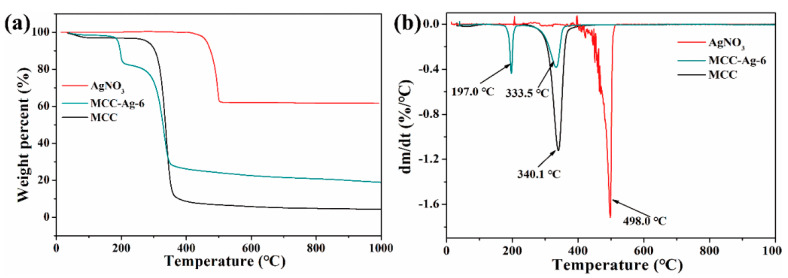
TGA (**a**)-DTG (**b**) of cellulose, AgNO_3_, and their mixture (MCC-Ag-6).

Figure 6 shows the TGA thermograms of cellulose with and without AgNO_3_ impregnation, together with that of neat AgNO_3_. The behaviour of MCC is typical of cellulose pyrolysis, showing a one-step weight loss between 300 °C and 360 °C via depolymerization into levoglucosan followed by secondary decomposition. The pyrolysis of neat AgNO_3_ gave a one-step weight drop of ca. 40% at between 420 °C and 450 °C. This decomposition is likely to result from the elimination of NO_2_ or NO.

The decomposition of the cellulose-AgNO_3_ mixture (MCC-Ag-6) is completely different from the superposition or intermediate of the two; the thermogram shows a sharp drop at 197 °C with a weight loss of 15%, suggesting a specific reaction between the AgNO_3_ and cellulose. Combined with the analysis of FT-IR and XRD, it can be concluded that after the treatment of MCC-Ag-6 at 150 ℃, a large number of the hydroxyl groups of MCC were oxidized to the carboxyl groups and the crystallinity of MCC was significantly reduced, which significantly decreased the thermal stability of the MCC. This may be the cause of the weight loss of the MCC-Ag-6 at 197 °C. As a result, the char yield from the MCC-Ag-6 was much higher than the pure cellulose, about 20% at 600 °C including silver.

**Figure 7:**
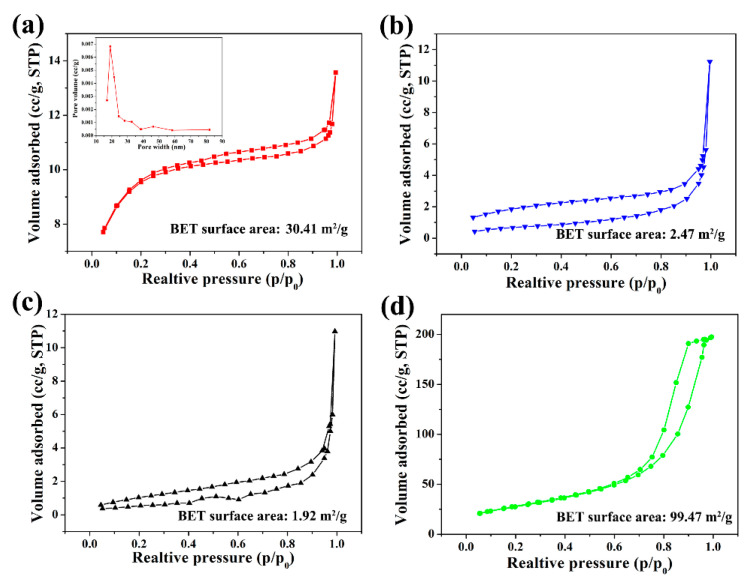
Nitrogen sorption/desorption isotherms. (**a**): MCC inset is pore size distribution; (**b**) MCC-Ag-6; (**c**) pyrolytic carbon from pure cellulose; (**d**) C-Ag-6.

Figure 7 shows the nitrogen sorption data. The specific surface areas of the MCC, MCC-Ag, AC and C-Ag-6 are 30.41, 2.47, 1.92 and 99.47 m^2^/g, respectively. The isotherm of MCC followed Type H4 of the IUPAC convention and was indicative of the presence of micropores and mesopores. Its pore size distribution mostly ranged 15–40 nm. The N_2_-sorption isotherms of the MCC-Ag and C-Ag-0 followed the IUPAC Type Ⅲ, indicating that the adsorbent–adsorbate interactions are relatively weak, and the surface of MCC-Ag and C-Ag-0 were nonporous or macroporous. This result indicates that the pores of the starting cellulose disappeared by pyrolysis and that the AgNO_3_ was embedded uniformly in the cellulose-derived carbon, not as nanoparticles deposited on the surface of cellulose.

Figure 7d shows the N_2_-sorption isotherm of C-Ag-6, the pyrolysis product. The isotherm Type H1 arose from the narrow pore size distribution of the mesoporous material, or the aggregates of relatively uniformly sized spherical particles. This feature confirms the morphology of the C-Ag-6, which was composed of uniformly sized silver particles and mesoporous carbon.

### 2.2. Size of Silver Nanosphere

**Figure 8:**
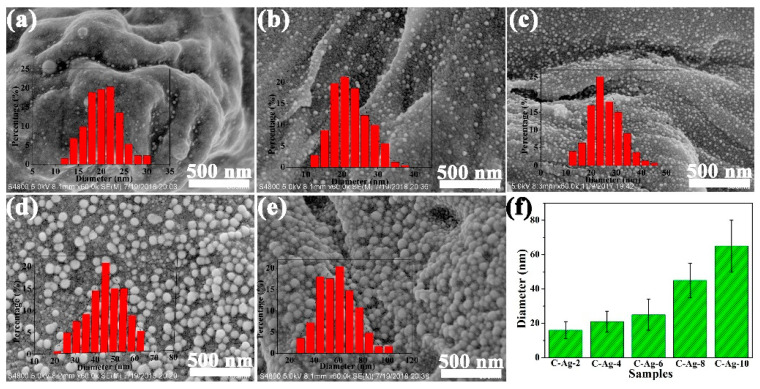
SEM images of (**a**) C-Ag-2; (**b**) C-Ag-4; (**c**) C-Ag-6; (**d**) C-Ag-8; (**e**) C-Ag-10 with particle size histogram as inset; (**f**) Median diameter. The particle size distribution determined by image analysis (sampling size: 500 particles).

Figure 8 shows the SEM of the carbon samples with varied silver content. Population and typical particle size increased with AgNO_3_ dose. The relative homogeneity of particle size depicted by the histograms is characteristic. This uniformity is likely to be a result of the restriction of the Ag atom in the cellulose–carbon matrix to form the spherical particles. At the maximum silver dose, silver particles completely covered the surface of the carbon matrix in the C-Ag-10 sample. Because of the nanoporous structure of MCC and strong interactions between silver ions and the carboxyl and hydroxyl groups of MCC, silver ions were firmly anchored to the MCC. Such interactions would lower the mobility of silver ions, enhance the formation of silver nuclei and prevent the growth of larger particles at low silver ion concentrations. At higher AgNO_3_ concentrations, higher amounts of silver ions are adsorbed in the MCC, resulting in large and widely distributed nanoparticles after pyrolysis.

**Figure 9:**
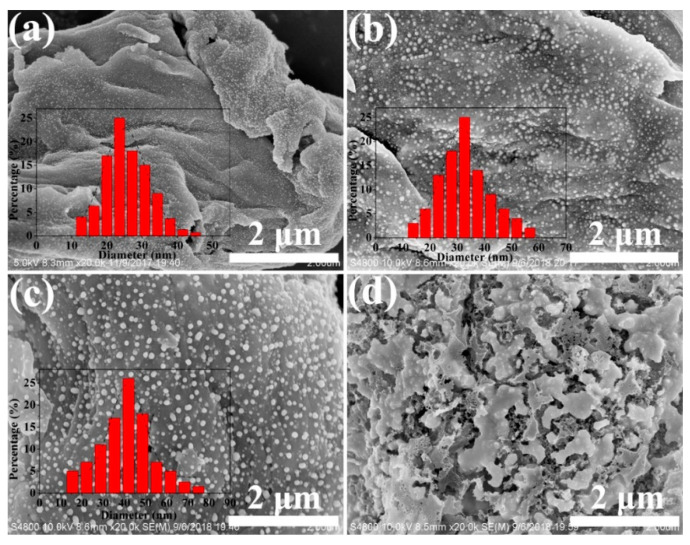
SEM images of C-Ag-6-600 (**a**); C-Ag-6-700 (**b**); C-Ag-6-800 (**c**); and C-Ag-6-900 (molten) (**d**). The particle size distribution is determined by image analysis (sampling size: 500 particles).

The morphology of the silver particles was strongly affected by temperature, as shown in Figure 9. The 600 °C treatment produced very small particles, and 700 °C and 800 °C produced increasingly larger particles. The mean diameter increased from 25 nm to 45 nm, and further increased at 800 °C. As the pyrolysis temperature increased, the size of the silver nanoparticles grew larger. This may be because high temperature is conducive to the crystallization of metallic silver, which causes the grain size to become larger. In the C-Ag-6-900 sample, the silver formed irregular block patterns (Figure 9d). This behaviour is apparently caused by the melting of silver. Though the temperature (900 °C) is lower than the melting point of silver (961 °C), the melting point of small particles is lowered by the surface effect. Some recently reported silver nanoparticles observed through different synthesis methods are listed in Table 1.

### 2.3. Electrical Property of Nanocomposite

**Figure 10:**
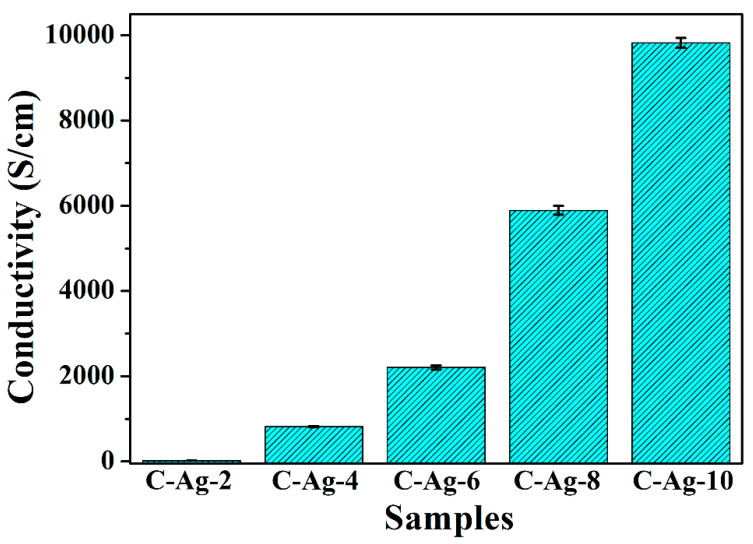
Conductivity of samples of C-Ag-2, C-Ag-4, C-Ag-6, C-Ag-8 and C-Ag-10.

Because metallic silver is highly conductive, the electrical properties of carbon composite with uniform-sized silver particles attracts attention. Figure 10 shows the electric surface conductivity of the pelleted samples measured using the four-probe method. The addition of silver raises conductivity sharply, reaching approx. 10^4^ S/cm for C-Ag-10. This behaviour is natural due to the difference between the conductivity of carbon and silver. The conductivity of the carbon without silver could not be determined due to the lack of tablet forming, but it is estimated at approx. 10^−3^ S/cm in the literature. In essence, the conductivity of the composites is caused by the evenly distributed silver particles. Compared with other similar biopolymer supports containing silver, the matrix carbon material can form a conductive path between the silver nanoparticles, so the conductivity of the C-Ag material is close to that of metal. The use of highly conductive carbon-based metal nanoparticles in electrode materials is also possible.

### 2.4. Antibacterial Property of Nanocomposite

**Figure 11:**
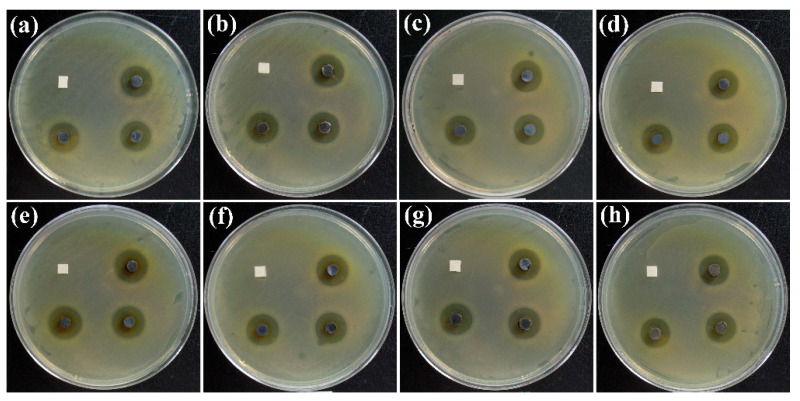
Inhibition zone of (**a**) C-Ag-2; (**b**) C-Ag-4; (**c**) C-Ag-8; (**d**) C-Ag-10; (**e**) C-Ag-6-600; (**f**) C-Ag-6-700; (**g**) C-Ag-6-800; and (**h**) C-Ag-6-900 against *E. coli*. The black tablets are the products; the white squares are filter paper pieces used as a control.

Silver nanomaterial is an effective antibacterial agent with wound-healing effects. The antibacterial activity of C-Ag-x for *E. coli* was measured using the disc diffusion method. Figure 11 shows that all C-Ag samples had an antimicrobial effect on *E. coli*. Table 2 lists the inhibition zone of the samples. The highest antimicrobial activities against *E. coli* were displayed on the C-Ag-6-600 sample, where the radius of the inhibition zone was 5.0 cm. Because MCC is transformed into carbon after pyrolysis, C-Ag exhibits superior stability compared to biopolymer supports containing silver, which makes it more practical for use under harsher conditions. Moreover, the silver nanoparticles were firmly embedded in the pores, which also meant that the C-Ag rarely precipitated silver nanoparticles during application, ensuring the biological safety of the application process.

## 3. Methods and Materials

### 3.1. Materials

Microcrystalline cellulose (MCC) was purchased from Sigma-Aldrich (St. Louis, MO, USA). Silver nitrate (AgNO_3_) was bought from Aladdin Reagent Co. (Shanghai, China) All of the analytical-grade reagents were used without further purification. Deionized water was used throughout.

### 3.2. Synthesis of Carbon-Supported Silver Nanospheres

Five grams of dry cellulose was dispersed in 100 mL of AgNO_3_ solution with concentration of 0.02–0.1 mol/L. The suspension was stirred at room temperature in the dark for 12 h and freeze-dried to form a white fluffy mass, denoted as MCC-Ag-x (x is 100 times the concentration in mol/L). The dry mass was pyrolyzed by an electric furnace under nitrogen from room temperature to 600 °C at 10 °C/min and kept there for 2 h. Products are denoted as C-Ag-x (x is 100 times of concentration in mol/L). Table 3 lists the conditions and the char yields. The effect of the maximum temperature on the size of the silver particles was tested for a 0.06 mol/L solution dose. The dry mass containing AgNO_3_ was pyrolyzed from room temperature to 700 °C, 800 °C or 900 °C. The products were named C-Ag-6-700, C-Ag-6-800 and C-Ag-6-900, respectively. 

The silver content was calculated using Equation (1):

(1) $$Ag\;content=\frac{m_{Ag}}{M}\times 100\%$$

where *m_Ag_* is the weight of the silver nanosphere, calculated from the amount of AgNO_3_ in the starting material of pyrolysis, and *M* is the weight of C-Ag product.

### 3.3. Characterization

The specific surface area and porous structure of the samples were determined by the nitrogen adsorption-BET method using an automated gas sorption analyser Quadrasorb SI-MP. Specific surface area and pore size distributions were analyzed according to the IUPAC Technical Report [50]. The morphological and elemental analysis were conducted using a Hitachi S-4800 equipped with an energy-dispersive X-ray spectroscope. Transmission electron microscopy was conducted with a JEOL JEM-2100 at 200 kV with a carbon film-coated grid. The sample was ground with a KBr with a weight ratio of 1:100. X-ray diffraction (XRD) was carried out with a Bruker D8 Focus with Cu Kα radiation (λ = 0.154 nm). Data were collected for 2θ = 5°–90° with a scanning step of 0.02. Electric conductivity was measured with the four-probe method and an RTS-9 electrometer (Four-probe Technologies Co., Guangzhou, China).

### 3.4. Antibacterial Test

Agar culture media (nutrient composition: 10.0 g of peptone, 5.0 g of beef extract, 5.0 g of NaCl, 15.0 g of agar, 1000 mL of distilled water adjust pH to 7.2–7.4) was coated with an aqueous suspension of Escherichia coli cells (106 CFU/mL), on which a C-Ag tablet (diameter: 0.5 cm) was inserted. For 16 to 18 h, the culture was incubated at 37 °C, and the mean radius of the inhibitory zone was determined.

## 4. Conclusions

A carbon–silver nanocomposite was produced via the pyrolysis of MCC with impregnated silver nitrate. The generation of silver nanoparticles with diameters of 10 to 100 nm were strongly aided by the swelling and oxidising of porous MCC by AgNO_3_, which was controlled by the pyrolysis temperature and silver dose. Additionally, this combination effectively fought against *E. coli* bacteria.

Nanoporous structure of MCC can be used as an effective nanoreactor for the in situ fabrication of silver nanoparticles. Pyrolysis of the MCC-AgNO_3_ mixture causes oxidation of MCC, leading to carboxyl groups, which not only anchor silver ions onto MCC but also stabilize silver nanoparticles through strong interactions with the surface silver atoms. Therefore, this research provides a new insight for designing size-controlled metallic nanoparticles and preparing a new functional cellulose nanocomposite.

## Figures and Tables

**Table 1:** Silver nanoparticles with different synthesis methods.

Composite	Template	Methods and Processes	Size Distribution (nm)	Ref.
AgNPs/PDA/graphitic-C_3_N_4_ nanocomposite	PDA/graphitic-C_3_N_4_	Sodium borohydride reduces silver nitrate	5–50	[28]
Ag nanoparticles	---	γ-radiation induced synthesis route	5–140, size controlled	[46]
Silver nanoparticles/cellulose nanocrystals	Cellulose nanocrystals	In situ chemical co-precipitation	5–40	[47]
Metal nanoparticles/cellulose gel	Cellulose gel	Adsorption and in situ reduction followed by pyrolysis	4–16, size controlled	[49]
Silver nanoparticles/pyrolytic carbon	Microcrystalline cellulose	Freeze drying and pyrolysis	10–100, size controlled	This study

**Table 2:** The inhibition zone of samples.

Samples	C-Ag-2	C-Ag-4	C-Ag-8	C-Ag-10	C-Ag-6-600	C-Ag-6-700	C-Ag-6-800	C-Ag-6-900
Inhibition zone (mm)	4.5	4.9	4.8	4.5	5.0	4.8	4.5	4.5

**Table 3:** Yield of pyrolytic carbon containing silver.

Start Sample	Cellulose (g)	AgNO_3_ (g)	AgNO_3_ Concentration (mol/L)	Result Sample	Char Yield (%)	Ag in Char (%) (Calc)
MCC-Ag-0	5.00	0.00	0.00	C-Ag-0	20.58	0.00
MCC-Ag-2	5.00	0.34	0.02	C-Ag-2	41.78	9.37
MCC-Ag-4	5.00	0.68	0.04	C-Ag-4	42.80	16.80
MCC-Ag-6	5.00	1.02	0.06	C-Ag-6	44.01	22.75
MCC-Ag-8	5.00	1.36	0.08	C-Ag-8	44.78	27.84
MCC-Ag-10	5.00	1.70	1.00	C-Ag-10	44.96	32.45

## Data Availability

Data will be available upon reasonable request from the corresponding author.
